# Machine Learning-Based Forecast of Hemorrhagic Stroke Healthcare Service Demand considering Air Pollution

**DOI:** 10.1155/2019/7463242

**Published:** 2019-11-03

**Authors:** Jian Chen, Hong Li, Li Luo, Yangyang Zhang, Fengyi Zhang, Fang Chen, Mei Chen

**Affiliations:** ^1^Business School, Sichuan University, Chengdu, Sichuan Province 610000, China; ^2^West China Hospital, Sichuan University, Chengdu, Sichuan Province 610000, China; ^3^The First People's Hospital of Longquanyi District, Chengdu, Sichuan Province 610100, China

## Abstract

This study aimed to forecast the pattern of the demand for hemorrhagic stroke healthcare services based on air quality and machine learning. Hemorrhagic stroke, air quality, and meteorological data for 2016-2017 were obtained from the Longquanyi District of China, and the study included 1932 cases. Six machine learning methods were used to forecast the demand for hemorrhagic stroke healthcare services considering seasonality and a lag effect, and the average area under the curve was as high as 0.7971. Our results indicate that (1) the performance of forecasting during the warm season is significantly better than that in the cold season, (2) considering air pollution would improve the performance of forecasting the demand for hemorrhagic stroke healthcare services using machine learning, (3) the association between the demand for hemorrhagic stroke healthcare services and air pollutants is linear to some extent, and (4) it is feasible to use short-term concentrations of air pollutants to forecast the demand for hemorrhagic stroke healthcare services. This practical forecast model could provide an advance warning regarding the potentially high numbers of hemorrhagic stroke admissions to medical institutions, thus allowing time to implement an appropriate response to the increase in patient volumes.

## 1. Introduction

Stroke, also known as cerebrovascular accident, cerebrovascular insult, or “brain attack,” occurs when poor blood flow to the brain results in cell death. From a statistical perspective, stroke is the second most common fatal disease in the world [1], the fourth most common disease in America [2], and the most common in China [3]. Thus, it is considered to seriously affect the physical health and quality of life of patients [4]. In 2013, 6.5 million patients who suffered from a stroke died, representing over a quarter of the number of stroke survivors (25.7 million) [1]. In addition, stroke imposes a significant economic burden on patients and healthcare services [5]. The total annual cost of stroke treatments in 2008 in the United States and European Union countries was estimated at $65.5 billion and €27 billion [6], respectively. In China, the annual cost of stroke care in 2011 was approximately RMB¥40 billion [3].

Factors affecting the clinical evolution of stroke include the physical condition of patients, such as the location of stroke [7], leukocyte level [8], and complications of stroke [9, 10]. In recent years, environmental health has continued to deteriorate with respect to air pollution, and smog from vehicular and industrial emissions has become a particular matter of concern for public and government policy. Simultaneously, the increasing prevalence of many diseases, including stroke, has increased the concern about air pollution as a serious threat to public health. Nitrogen dioxide (NO_2_) and particulate matter with an aerodynamic diameter of r10 *μ*m (PM_10_) are significantly associated with cardiovascular mortality, with increasing concentrations of NO_2_ noted to have a greater impact on cardiovascular mortality among men and the elderly [11]. Pearce et al. [12] showed that exposure to high levels of outdoor nitrogen oxide is significantly associated with an increased risk of stroke. Wing et al. [13] revealed that higher levels of particulate matter with a grain size of 2.5 *μ*m or less (PM_2.5_) and ozone (O_3_) are associated with a higher incidence of stroke. Several epidemiological studies [14, 15] have reported a significant positive correlation between air pollution and stroke. PM_2.5_, NO_2_, PM_10_, carbon monoxide (CO), and O_3_ are the most common pollutants associated with stroke.

As a core component of health systems, healthcare service management aims to notify the related institutions of the expected demand in a timely and accurate fashion, enabling these institutions to make effective decisions on resource allocation and reinforce their healthcare systems for the anticipated demand [16]. Particularly, Liu et al. [17] also demonstrated that short-term exposure to PM_2.5_ and PM_10_ increased the risk of hemorrhagic stroke, which accounts for 15% of all stroke cases and 40% of deaths due to stroke. Hence, the key to optimizing healthcare resource allocation and improving the quality of health services is to forecast the possible excess demand for stroke healthcare services, especially that of hemorrhagic stroke, according to changes in external environmental factors, such as air quality.

To the best of our knowledge, few studies have focused on forecasting the demand for stroke healthcare services. However, many studies have used machine learning to forecast the effect of air quality on diseases. Soyiri et al. [16] utilized a multistage quantile regression approach to forecast the excess demand for healthcare services in the form of daily asthma admissions by using retrospective data on weather and air quality from the Hospital Episode Statistics database. Moustris et al. [18] developed three different artificial neural network models to forecast the total weekly number of childhood asthma admissions in the greater Athens area of Greece. Three different artificial neural network models were developed and trained to forecast childhood asthma admissions for subgroups of 0–4- and 5–14-year-olds as well as the entire study population. Using data regarding weather factors, air quality, and hospital asthma admissions, Soyiri et al. [16] developed two related negative binomial models to forecast admissions due to asthma in London. Zhang et al. [19] analyzed and forecasted the monthly hospital admissions and hospitalization expenses for respiratory diseases in Shanghai using the autoregressive integrated moving average model. These studies indicate that machine learning (including traditional statistical learning) can be used to forecast such issues. However, these studies used only a single method to forecast healthcare service demand and did not conduct comparative analysis to determine the proper model in forecasting. In addition, feature selection, which may help facilitate the forecast process, was not considered.

In addition, seasonality is also an important factor. Zhang et al. [20] indicated that seasonal patterns in health impacts of air pollution have been demonstrated in a number of previous investigations, whereas findings were less consistent, with peaks occurring in cold, hot, or transitional seasons. The China Air Pollution and Health Effects Study (CAPES) identified a two-peak (winter and summer) seasonal pattern in 17 Chinese cities for PM_10_-related mortality effect. Also, the season-modified effects varied by geographic regions in several Chinese single-city investigations. In addition, Xiang et al. [21] demonstrated that, in contrast to the warm season, NO_2_ concentrations were significantly correlated with stroke hospitalization rates during the cold season. Hence, it is more befitting to construct different forecast models for different seasonal patterns.

This study aimed to forecast the pattern of the demand for hemorrhagic stroke healthcare services based on air quality using machine learning techniques. Due to the disparity in the association between the demand for hemorrhagic stroke healthcare services and air quality in different seasons, we constructed two different forecast models. In addition, a lag effect was also considered in selecting features for forecasting and the model with optimal performance. This practical forecast model could provide advance warning to medical institutions. Healthcare resource managers can also allocate the corresponding resources according to the expected demand, thus guaranteeing the accessibility of timely healthcare resources. Based on our research, a surveillance system to enhance early detection and interventions for hemorrhagic stroke can be implemented in advance to avoid shortages in healthcare resources due to hemorrhagic stroke.

## 2. Data and Experiment Setup

### 2.1. Data

Data regarding hemorrhagic stroke events for 2016-2017 were obtained from medical records sourced from the Center for Disease Control and Prevention in the Longquanyi District of China, a 55,698 ha area with a population of approximately 643,000 in southeast Chengdu. The dataset included 7,230 stroke events; among them, there were 1932 cases of hemorrhagic stroke. Because nearly all the medical data for the region are recorded at this center, the data can be considered as representative of hemorrhagic stroke occurrence across the entire population of the Longquanyi District. Within these data, the personal information of deceased patients was recorded, including the date of hemorrhagic stroke onset and demographics.

Data regarding air pollution for the period 2016-2017 were obtained from environmental monitoring stations in the Longquanyi District of Chengdu including data regarding the concentrations of PM_2.5_, PM_10_, CO, NO_2_, O_3_, and sulfur dioxide (http://www.cnemc.cn/). All data regarding air quality were recorded in kilograms per cubic meter but converted into milligrams per cubic meter for CO and parts per million for the other pollutants. Since temperatures may affect the incidence of stroke [22], the minimum and maximum daily temperatures recorded by the Longquanyi District Meteorological Agency were also used as predictors. This study did not involve human subjects and adhered to all current laws of China.

To identify seasonal disparities, and considering that Chengdu is located in Southwest China with a subtropical monsoon climate, we distinguished between warm and cold seasons. The period between April 1 and September 30 was regarded as the warm season, while all other months were regarded as the cold season.

### 2.2. Experiment Setup

Our study views the pattern of the demand for hemorrhagic stroke healthcare services in Longquanyi District as a complex and nonlinear system and assumed that the newly occurred hemorrhagic stroke events would have no effects on the system.

Data analysis was performed in 2 stages: a descriptive statistical process and forecast process. In the former stage, we performed descriptive statistical analyses of air pollution data and historical data. Population stroke status included two: “normal” and “excess.” “Normal” referred to a scenario in which the number of stroke events on a certain day was lower than the capacity limit, while “excess” referred to a scenario in which the number of stroke events was higher than the capacity limit. In our study, the capacity limit was defined as the number of events that covered 70% of the demand for hemorrhagic stroke healthcare services.

In the forecast process, data regarding daily hemorrhagic stroke admissions, minimum and maximum daily temperature, and air quality were merged by date to form a time-series dataset. Lag effects were also considered in this study. The lag of a scheme, *N*, is considered when the data from the preceding day to *N* days prior are used. For each scheme, the lag varied from 1 to 14. In order to abstract the key feature, we used the least absolute shrinkage and selection operator (LASSO) regression to simplify the model and determine the risk factor sets considering lag effects, considering that LASSO is a good solution to avoid multicollinearity of air pollutants. Ten-fold cross-validation was used to retain the reliable and stable model. MaxLag-N refers to the risk factor sets that considered the air quality variables of the recent N days.

The select subsets of MaxLag-N were used to train and test machine learning models with 10-fold cross-validation. The following machine learning models were considered in our study: logistic regression (LR), random forest (RF), support-vector machines with linear kernel (SVMLinear), k-nearest neighbor algorithm (KNN), and extreme gradient boosting decision tree (XGBTree) and extreme gradient boosting linear (XGBLinear) models, which are extreme gradient boosting algorithms based on tree and linear models, respectively.

The evaluation metrics included the area under the curve (AUC), sensitivity, and specificity. The larger the AUC value, the better the model distinguishes the prediction target ability and the better the overall model prediction effect. Sensitivity refers to the proportion of actual high-incidence prediction targets that are predicted to be high-risk prediction targets. Specificity refers to the proportion of the actual low-incidence prediction targets that are predicted to be low-incidence targets.

In this study, we first partitioned the dataset into warm and cold datasets according to the date of hemorrhagic stroke onset. Then, MaxLag-N (*N* arranged from 1 to 14) risk factor sets of warm and cold datasets were determined by Lasso regression, respectively, and the models considering different *N* values and datasets using the aforementioned machine learning methods were trained and tested. *Moreover, the models without considering air pollution were also trained; the performances of them were also analyzed, and comparative analysis against air pollution situation was also conducted*. Finally, statistical tests were performed to assess the disparities in the performance (especially AUC) with respect to seasons, lags, and machine learning models.

## 3. Results

During the study period, the daily average number of hemorrhagic stroke events was 2.9861 (standard deviation (SD), 1.8650). During the warm season, the daily average number of hemorrhagic stroke events was 2.9780 (SD, 1.9848), and there were a total of 947 hemorrhagic stroke events. During the cold season, the daily average number of hospital admissions due to hemorrhagic stroke was 2.9939 (SD, 1.7443), and there were a total of 985 hemorrhagic stroke events. Hence, compared to the large population (approximately 643,000 residents), the newly occurred hemorrhagic stroke events (averagely 2.9861 cases per day) would have no effects on the system, which indicates that the assumption in this study is reasonable.  Table 1 also shows the related statistics in detail.

Mean denotes the average number of daily hemorrhagic stroke events. SD denotes the standard deviation of the number of hemorrhagic stroke events. Min and Max denote the minimum and maximum number of hemorrhagic events, respectively, and Sum denotes the sum of different hemorrhagic stroke events. Each quartile of the daily events is shown under the respective percentage.

Table 2 shows the daily level of different atmospheric pollutants, including the average daily level in the research period (2015-12-17 to 2017-12-31), the SD of the daily average concentration of each air pollutant, and the highest daily level of different atmospheric pollutants (Max). The main atmospheric pollutants were PM_2.5_ and O_3_; these were the main pollutants on up to 720 days (of a total of 989 days).

**Figure 1:**
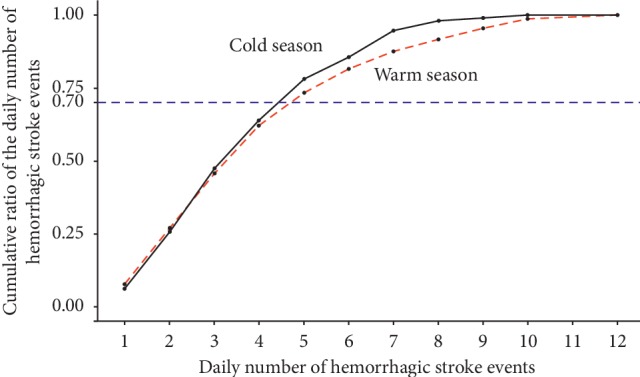
The curves of the cumulative proportion of hemorrhagic stroke event counts in the cold and warm seasons.

To define the population hemorrhagic stroke healthcare demand status, we assessed the total number of hemorrhagic stroke events to identify the threshold of the daily population hemorrhagic stroke status during the warm and cold seasons.  Figure 1 describes the hemorrhagic stroke events of each day and presents a homogeneous degree of hemorrhagic stroke events for daily hemorrhagic stroke event counts. The *x*-axis denotes the daily number of hemorrhagic stroke events, and the *y*-axis denotes the cumulative proportion of hemorrhagic stroke events. The black solid and red dashed curves denote the daily hemorrhagic stroke event counts in the cold and warm seasons, respectively. Hence, the threshold daily numbers of hemorrhagic stroke cases in the cold and warm seasons were 4 and 5, according to the “nearest” criteria.

According to the study design, all data were partitioned into the warm and cold datasets according to the date of hemorrhagic stroke onset. Then, MaxLag-N (with N arranged from 1 to 14) risk factor sets of warm and cold datasets were determined by Lasso regression, respectively. The models considered different *N* values, and the datasets using the aforementioned machine learning methods were trained and tested. Comparative analysis between the warm and cold seasons was performed using the *t*-test.  Table 3 shows the results of the comparative analysis and presents the *P* values of the *t*-test and the average values of the evaluation metrics. The average AUC of the models for the warm season was 0.6801, while the average AUC of the models of the cold season was 0.5721. There were significant differences in all evaluation metrics between the warm and cold seasons. In addition, the performances of the models for the cold season were not good enough (AUC: 0.5721); hence, we focused only on the models for the warm season in the subsequent analyses. In addition, the risk factor sets of warm datasets selected by LASSO are shown in Table 4.

Table 5 shows the statistics on the performance of the models for the warm season according to the machine learning methods. LR was the most effective model and had the best performance (mean AUC, 0.7369; SD, 0.0276); the other models performed inferiorly to LR and had average AUC values >0.65. The models used, in decreasing order of average AUC, were LR, RF, SVMLinear, KNN, XGBLinear, and XGBTree. LR also had the highest sensitivity (0.4684) and specificity (0.8708). Apart from LR, the other models all had average sensitivities <0.3. The models used, in order of average sensitivity, were LR, XGBLinear, KNN, RF, XGBTree, and SVMLinear. Apart from SVMLinear (average specificity, 0.7483), the other models all had average specificities >0.80. The other models used, in decreasing order of average specificity, were LR, XGBTree, KNN, XGBLinear, and RF.

Table 6 shows the *P* values of the *t*-test between different machine learning methods regarding AUC. The null assumption of the *t*-test is that there are no significant differences between different machine learning methods. The *P* value refers to the risk of wrongly rejecting the null assumption. If the *P* value is less than 0.05, we would prefer to reject the null assumption due to the low risk of making an error; otherwise, we would prefer to accept the null assumption. As shown in Table 6, there were significant differences between LR and all other models at the 0.001 significance level. In addition, the difference between XGBTree and RF was also significant, but at the 0.05 significance level.

In addition, Table 7 presents the performance of warm season models without considering air pollution among the machine learning methods. In Table 7, the mean value of AUC of LR, RF, SVMLinear, and XGBTree without considering air pollution is lower than that considering air pollution; but, for KNN and XGBLinear, the situation is quite opposite. When air pollution was not taken into consideration, RF, SVMLinear, KNN, and XGBLinear performed better in the aspect of sensitivity, and in the aspect of specificity, RF, SVMLinear, and XGBLinear performed better, respectively. However, the standard deviations of all three metrics for all models without considering air pollution are higher than that considering air pollution.  Table 8 shows the *P* values of the *t*-test between models with and without considering air pollution regarding different metrics. According to Table 8, only in two scenarios the difference between with and without considering air pollution is significant: LR with AUC and SVMLinear with specificity.

Table 9 shows the statistics of the performance of warm season models in terms of lag effects. The best lag period was MaxLag-14, considering not only the average AUC of MaxLag-14 but also other evaluation indexes (AUC, 0.7314). A different effect was found in the accuracy of prediction when different lag days were considered.

Table 10 shows the models with AUC >0.75. The best model in our study was LR considering a 14-day lag effect, and its AUC (0.7971) was much closer to 0.8. This model in particular was the best model and had the best lag. In addition, four other models had AUC >0.75: SVMLinear with MaxLag-14, LR with MaxLag-13, RF with MaxLag-14, and LR with MaxLag-9.

## 4. Discussion

This study aimed to forecast the pattern of the demand for hemorrhagic stroke healthcare services based on air quality using machine learning that considered lag effect and season disparity. A few insights in the aspects of feasibility, model selection, and season disparity are presented below.

LR achieves the best performance in both air pollution situation and nonair pollution situation in the aspect of AUC. In addition, the difference between the two situations for LR is significant in the aspect of AUC. Hence, according to the results, air pollution has a positive effect on forecasting hemorrhagic stroke healthcare service demand.

It is feasible to use short-term concentrations of air pollutants to forecast the demand for hemorrhagic stroke healthcare services. In our study, we used only pollution information from up to 14 days to forecast the demand for hemorrhagic stroke healthcare, and it achieved a good level of performance. For MaxLag-14 models, the average AUC was 0.7314. For LR with MaxLag-14 in particular, the average AUC was as high as 0.7971. This AUC value was approximately 0.8 and could yield great effects in practical implementation.

Among all machine learning methods, the linear models achieved the best performance. In general, the average AUC of the linear models (LR, SVMLinear, and XGBLinear) were better than that of the other models (RF, KNN, and XGBTree). LR, the most commonly used linear model, achieved the best performance in all aspects (AUC, sensitivity, and specificity). These results may indicate that the association between the demand for hemorrhagic stroke healthcare services and air pollutants is linear to some extent.

The performance of forecasting during the warm season was significantly better than that during the cold season. The average AUC, sensitivity, and specificity of the warm season were higher than those of the cold season, and the *P* values of the *t*-test were all <0.0001, which indicate that the warm season models were significantly superior to the cold season models. In a study conducted by Xiang et al. [21], NO_2_ concentrations were significantly correlated with stroke hospitalization rates during the cold season rather than the warm season. According to Xiang et al. [21], an intuitive inference can be given: the cold season models were significantly superior to the warm season models, and this is in contrast to our results. This disparity may lie in the fact that Xiang et al. [21] considered only a single air pollutant, while we considered six air pollutants and temperature simultaneously.

Our study has some limitations. Although most representative machine learning techniques were considered in this study, the number of machine learning techniques was still limited. In addition, although Lasso is well acknowledged as a useful feature selection method, other feature selection methods should also be considered. Finally, this research involved only hemorrhagic stroke events that occurred in a single region. Regional disparities may exist in terms of performance. Further comparative research will be conducted to support the findings of the present study and address potential disparities.

## 5. Conclusions

We developed a practical city-based forecast model using machine learning methods and the concentration of air pollutants. The results of our study indicate that (1) the performance of forecasting in the warm season is significantly better than that in the cold season, (2) considering air pollution would improve the performance of forecasting the demand for hemorrhagic stroke healthcare services using machine learning, (3) the association between the demand for hemorrhagic stroke healthcare services and air pollutants is linear to some extent, and (4) it is feasible to use short-term concentrations of air pollutants to forecast the demand for hemorrhagic stroke healthcare services. This practical forecast model could provide warnings in advance to medical institutions regarding the potentially high numbers of admissions due to hemorrhagic stroke, thus allowing time to implement an appropriate response to the increase in patient volumes.

## Figures and Tables

**Table 1:** Statistics on the incidence of hemorrhagic stroke.

Duration	Mean	SD	Min	25%	50%	75%	Max	Sum
All	2.9861	1.8650	0	2	3	4	12	1932
Warm season	2.9780	1.9848	0	2	2	4	12	947
Cold season	2.9939	1.7443	0	2	3	4	10	985

**Table 2:** Statistics on air pollution and temperature.

Pollutants	Mean	SD	Min	25%	50%	75%	Max
SO_2_	11.0584	5.9144	3	7	9	13	46
NO_2_	45.5800	21.6757	12	28	43	58.5	121
CO	1.0719	0.5262	0.4	0.8	0.9	1.2	10
O_3_	98.7302	55.9866	2	55	92	136	334
PM_2.5_	57.5307	43.9921	4	27	44	75	287
PM_10_	90.5947	63.6597	6	47	71	113.75	411
Lowest	21.6461	7.8395	5	14	22	29	36
Highest	14.3133	7.2099	–4	7	15	20	26

Mean denotes the average daily concentration. SD denotes the standard deviation of concentration. Min and Max denote the minimum and maximum concentrations; each quartile of the concentration is shown under the respective percentage. Lowest and highest denote the minimum and maximum temperatures, respectively.

**Table 3:** Comparative analysis of the performances of the models for the cold and warm seasons.

	AUC	Sensitivity	Specificity
Cold season	0.5721	0.1689	0.6934
Warm season	0.6801	0.2778	0.8353
*P* values	<0.0001	<0.0001	<0.0001

*P* values were obtained from the *t*-test between the performances in the cold and warm seasons.

**Table 4:** The risk factors of warm datasets selected by LASSO.

MaxLag	Elements
1	PM_10__1, CO_1, Low_1
2	PM_10__1, CO_1, CO_2, Low_1, Low_2
3	CO_1, CO_3, Low_3
4	CO_1, CO_4, Low_3
5	CO_1, CO_4, Low_1, Low_3
6	CO_1, CO_4, Low_3
7	CO_1, CO_4, Low_3
8	CO_1, CO_4, Low_3, Low_8
9	CO_1, CO_4, Low_3, Low_8
10	CO_1, CO_4, Low_3, Low_8
11	CO_1, CO_4, Low_3, Low_8
12	CO_1, CO_4, Low_3, Low_8
13	PM_10__12, PM10_13, CO_1, CO_4, CO_13, Low_3, Low_8
14	PM_10__13, CO_4, CO_14, Low_3, Low_8

Suffix “_*N*” denotes the lag of *N*; for example, CO_1 refers to the concentration of CO one day ago. Low refers to lowest temperature.

**Table 5:** Statistics on the performance of warm season models considering air pollution among the machine learning methods.

Model	M-AUC	M-Sens	M-Spec	SD-AUC	SD-Sens	SD-Spec
LR	0.7369	0.4684	0.8708	0.0276	0.0687	0.0137
RF	0.6811	0.2520	0.8368	0.0434	0.1096	0.0068
SVMLinear	0.6743	0.1795	0.7483	0.0409	0.0467	0.1159
KNN	0.6681	0.2558	0.8551	0.0371	0.1312	0.0224
XGBLinear	0.6601	0.2915	0.8448	0.0329	0.0574	0.0068
XGBTree	0.6599	0.2195	0.8563	0.0373	0.0838	0.0126

M-AUC, M-Sens, and M-Spec denote the average area under the curve (AUC), sensitivity, and specificity, respectively; SD-AUC, SD-Sens, and SD-Spec denote the standard deviation of the AUC, sensitivity, and specificity, respectively. LR, logistic regression; RF, random forest; SVMLinear, support-vector machines with linear kernel; KNN, k-nearest neighbor algorithm; XGBTree, extreme gradient boosting decision tree; XGBLinear, extreme gradient boosting linear model.

**Table 6:** The *P* values of the *t*-test between different machine learning methods regarding AUC.

	LR	XGBTree	XGBLinear	KNN	SVMLinear	RF
LR	1	<0.0001^*∗∗∗*^	<0.0001^*∗∗∗*^	0.0001^*∗∗∗*^	<0.0001^*∗∗∗*^	<0.0001^*∗∗∗*^
XGBTree	<0.0001^*∗∗∗*^	1	0.9878	0.5949	0.1579	0.1265
XGBLinear	<0.0001^*∗∗∗*^	0.9878	1	0.5846	0.2964	0.0326^*∗*^
KNN	0.0001^*∗∗∗*^	0.5949	0.5846	1	0.6933	0.4353
SVMLinear	<0.0001^*∗∗∗*^	0.1579	0.2964	0.6933	1	0.5933
RF	<0.0001^*∗∗∗*^	0.1265	0.0326^*∗*^	0.4353	0.5933	1

^*∗∗∗*^0.001, ^*∗∗*^0.01, and ^*∗*^0.05. LR, logistic regression; RF, random forest; SVMLinear, support-vector machines with linear kernel; KNN, k-nearest neighbor algorithm; XGBTree, extreme gradient boosting decision tree; XGBLinear, extreme gradient boosting linear model.

**Table 7:** Statistics on the performance of warm season models without considering air pollution among the machine learning methods.

Model	M-AUC	M-Sens	M-Spec	SD-AUC	SD-Sens	SD-Spec
LR	0.6062–	0.4137–	0.8543–	0.1452+	0.3688+	0.0367+
RF	0.6504–	0.3750+	0.8571+	0.1011+	0.4361+	0.0444+
SVMLinear	0.6229–	0.2996+	0.8217+	0.1004+	0.2943+	0.0812–
KNN	0.6931+	0.3667+	0.8510–	0.1173+	0.4830+	0.0469+
XGBLinear	0.6783+	0.3600+	0.8590+	0.1415+	0.3719+	0.0428+
XGBTree	0.6453–	0.3333–	0.8422–	0.0881+	0.4157+	0.0258+

M-AUC, M-Sens, and M-Spec denote the average area under the curve (AUC), sensitivity, and specificity, respectively; SD-AUC, SD-Sens, and SD-Spec denote the standard deviation of the AUC, sensitivity, and specificity, respectively. LR, logistic regression; RF, random forest; SVMLinear, support-vector machines with linear kernel; KNN, k-nearest neighbor algorithm; XGBTree, extreme gradient boosting decision tree; XGBLinear, extreme gradient boosting linear model. “+” indicates that the corresponding value without considering air pollution is higher than that considering air pollution. “–” indicates that the corresponding value considering air pollution is higher than that without considering air pollution.

**Table 8:** The *P* values of the *t*-test between models with and without considering air pollution regarding different metrics.

	XGBTree	XGBLinear	LR	KNN	SVMLinear	RF
AUC	0.2114	0.3777	0.0316^*∗*^	0.969	0.3872	0.6214
Sensitivity	0.7939	0.0748	0.2558	0.2202	0.267	0.5365
Specificity	0.1211	0.1897	0.2148	0.4136	0.0004^*∗∗∗*^	0.5913

^*∗∗∗*^0.001, ^*∗∗*^0.01, and ^*∗*^0.05. LR, logistic regression; RF, random forest; SVMLinear, support-vector machines with linear kernel; KNN, k-nearest neighbor algorithm; XGBTree, extreme gradient boosting decision tree; XGBLinear, extreme gradient boosting linear model.

**Table 9:** Statistics on the performance of warm season models regarding lag effects.

Lag	M-AUC	M-Sens	M-Spec	SD-AUC	SD-Sens	SD-Spec
MaxLag-14	0.7314	0.3524	0.8040	0.0568	0.1631	0.1360
MaxLag-9	0.6961	0.2863	0.8574	0.0369	0.1329	0.0184
MaxLag-6	0.6948	0.3205	0.8584	0.0367	0.1383	0.0215
MaxLag-11	0.6892	0.2902	0.8266	0.0309	0.1096	0.0763
MaxLag-13	0.6876	0.2671	0.8010	0.0455	0.1619	0.1286
MaxLag-10	0.6855	0.2644	0.8534	0.0356	0.1318	0.0170
MaxLag-8	0.6803	0.2399	0.8422	0.0440	0.1273	0.0342
MaxLag-4	0.6790	0.2953	0.8372	0.0310	0.1407	0.0359
MaxLag-3	0.6785	0.2168	0.8231	0.0287	0.1443	0.0548
MaxLag-12	0.6754	0.2643	0.8246	0.0528	0.1056	0.0769
MaxLag-5	0.6742	0.2680	0.8477	0.0530	0.1080	0.0127
MaxLag-7	0.6683	0.3191	0.8289	0.0541	0.1564	0.0767
MaxLag-1	0.6409	0.3038	0.8486	0.0350	0.0882	0.0121
MaxLag-2	0.6396	0.2008	0.8417	0.0371	0.0746	0.0066

M-AUC, M-Sens, and M-Spec denote the average area under the curve (AUC), sensitivity, and specificity, respectively; SD-AUC, SD-Sens, and SD-Spec denote the standard deviation of the AUC, sensitivity, and specificity, respectively. MaxLag-N refers to the risk factor sets that considered the air quality variables of the recent *N* days.

**Table 10:** Performance of warm season models with AUC >0.75

Lag	Models	M-AUC	M-Sens	M-Spec	SD-AUC	SD-Sens	SD-Spec
MaxLag-14	LR	0.7971	0.6252	0.8929	0.1158	0.2802	0.0429
MaxLag-14	SVMLinear	0.7741	0.2266	0.5293	0.0829	0.2942	0.4593
MaxLag-13	LR	0.7588	0.5483	0.8963	0.1289	0.2789	0.0541
MaxLag-14	RF	0.7567	0.3500	0.8489	0.1116	0.4191	0.0307
MaxLag-9	LR	0.7549	0.4617	0.8707	0.0668	0.2632	0.0347

M-AUC, M-Sens, and M-Spec denote the average area under the curve (AUC), sensitivity, and specificity, respectively; SD-AUC, SD-Sens, and SD-Spec denote the standard deviation of the AUC, sensitivity, and specificity, respectively. MaxLag-N refers to the risk factor sets that considered the air quality variables of the recent *N* days. LR, logistic regression; RF, random forest; SVMLinear, support-vector machines with linear kernel; KNN, k-nearest neighbor algorithm; XGBTree, extreme gradient boosting decision tree; XGBLinear, extreme gradient boosting linear model.

## Data Availability

The data supporting the findings of this study will not be shared since it is an organizational property. Data were anonymous, and study subjects could not be identified.
